# C_2_HEST score predicts clinical outcomes in heart failure with preserved ejection fraction: a secondary analysis of the TOPCAT trial

**DOI:** 10.1186/s12916-021-01921-w

**Published:** 2021-02-18

**Authors:** Weihao Liang, Yuzhong Wu, Ruicong Xue, Zexuan Wu, Dexi Wu, Jiangui He, Yugang Dong, Gregory Y. H. Lip, Wengen Zhu, Chen Liu

**Affiliations:** 1 Department of Cardiology, The First Affiliated Hospital of Sun Yat-Sen University, Guangzhou, 510080 People’s Republic of China; 2 NHC Key Laboratory of Assisted Circulation (Sun Yat-sen University), Guangzhou, 510080 People’s Republic of China; 3 National-Guangdong Joint Engineering Laboratory for Diagnosis and Treatment of Vascular Diseases, Guangzhou, People’s Republic of China; 4 Liverpool Centre for Cardiovascular Sciences, Institute of Ageing and Chronic Disease, University of Liverpool, Liverpool, UK; 5 Aalborg Thrombosis Research Unit, Department of Clinical Medicine, Aalborg University, Aalborg, Denmark

**Keywords:** Heart failure, Atrial fibrillation, Risk prediction, Outcomes

## Abstract

**Background:**

The C_2_HEST score has been validated for predicting AF in the general population or post-stroke patients. We aimed to assess whether this risk score could predict incident AF and other clinical outcomes in heart failure with preserved ejection fraction (HFpEF) patients.

**Methods:**

A total of 2202 HFpEF patients without baseline AF in the TOPCAT trial were stratified by baseline C_2_HEST score. Cox proportional hazard model and competing risk regression model was used to explore the relationship between C_2_HEST score and outcomes, including incident AF, stroke, all-cause death, cardiovascular death, any hospitalization, and HF hospitalization. The discriminative ability of the C_2_HEST score for various outcomes was assessed by calculating the area under the curve (AUC).

**Results:**

The incidence rates of incident AF, stroke, all-cause death, cardiovascular death, any hospitalization, and HF hospitalization were 1.79, 0.70, 3.81, 2.42, 15.50, and 3.32 per 100 person-years, respectively. When the C_2_HEST score was analyzed as a continuous variable, increased C_2_HEST score was associated with increased risk of incident AF (HR 1.50, 95% CI 1.29–1.75), as well as increased risks of all-cause death, cardiovascular death, any hospitalization, and HF hospitalization. The AUC for the C_2_HEST score in predicting incident AF (0.694, 95% CI 0.640–0.748) was higher than all-cause death, cardiovascular death, any hospitalization, or HF hospitalization.

**Conclusions:**

The C_2_HEST score could predict the risk of incident AF as well as death and hospitalization with moderately good predictive abilities in patients with HFpEF. Its simplicity may allow the possibility of quick risk assessments in busy clinical settings.

**Supplementary Information:**

The online version contains supplementary material available at 10.1186/s12916-021-01921-w.

## Background

Heart failure with preserved ejection fraction (HFpEF) is a highly complex clinical syndrome with a high prevalence that increases with age. HFpEF and atrial fibrillation (AF) have many shared risk factors, and thus, they are intertwined disorders and often coexist in clinical settings [1]. Epidemiological studies have suggested that HFpEF patients are at an increased risk of AF [2], whereas AF is associated with increased risks of adverse cardiovascular events in HFpEF patients [3]. Therefore, HFpEF patients should be screened for AF to prevent adverse cardiovascular events, and early identification of those HFpEF patients at risk of AF may prompt the initiation of stroke prevention treatment and thus improve prognosis. Several studies have proposed a series of risk scoring models for predicting adverse outcomes such as stroke [4] and death [5] among HFpEF patients. However, a clinical risk score for predicting AF in patients with HFpEF remains to be established.

The use of a clinical risk stratification score may facilitate targeted efforts to intensify screenings in subjects at high risk of developing AF. A prior systematic review has summarized ten risk scoring models specifically used for predicting incident AF in the general population [6]. Within these established risk scoring systems, the C_2_HEST score is the latest and simplest one, which has been derived from a large cohort of 471,446 Chinese subjects and validated in a cohort of 514,764 Korean subjects in the community [7]. Subsequently, the predictive ability of the C_2_HEST score for incident AF has been validated in the healthy Danish population [8] and post-stroke European patients [9]. However, the predictive ability of the C_2_HEST score for stratifying the AF risk has not been previously determined in patients with HFpEF.

Associations of individual components in the C_2_HEST score with adverse outcomes have previously been established in patients with HFpEF. However, whether the C_2_HEST score could predict clinical outcomes such as death and HF hospitalization in patients with HFpEF is still unknown. In the present study, based on the data from the Treatment of Preserved Cardiac Function Heart Failure with an Aldosterone Antagonist (TOPCAT) trial [10], we performed a post hoc analysis of HFpEF patients to assess the diagnostic performance of the C_2_HEST score for incident AF as well as other clinical outcomes including stroke, death, and hospitalization.

## Methods

### Study subjects

We acquired the dataset of the TOPCAT trial (phase III, randomized, double-blind, placebo-controlled) from the National Heart, Lung, and Blood Institute. The dataset of this trial was obtained from the National Heart, Lung, and Blood Institute (NHLBI) by applying to Biologic Specimen and Data Repository Information Coordinating Center (BIOLINCC, https://biolincc.nhlbi.nih.gov/). Our study was approved by the Medical Ethical Committee of the First Affiliated Hospital, Sun Yat-sen University. The TOPCAT investigators were not involved in our current study.

The study design of the TOPCAT trial had been reported previously [11]. A total of 3445 patients from the Americas (USA, Canada, Brazil, and Argentina) and Russia/Georgia, with an age of ≥ 50 years, a left ventricular ejection fraction (LVEF) of ≥ 45%, a serum potassium level of < 5.0 mmol/L, and a history of HF hospitalization within the previous 12 months or elevated brain natriuretic peptide level within 60 days before randomization were enrolled. Exclusion criteria included severe systemic illness with a life expectancy of less than 3 years, severe renal dysfunction, and specific coexisting conditions.

### Risk stratification using the C_2_HEST score

In this study, we included 2202 patients who were without AF at baseline including a history of AF or AF confirmed on an electrocardiogram (ECG) at enrollment. Variables of patient characteristics at baseline were retrieved from the dataset to calculate the C_2_HEST score with a total of 6 individual components including coronary artery disease (1 point), chronic obstructive pulmonary disease (COPD, 1 point each), hypertension (1 point), elderly (age ≥ 75 years, 2 points), systolic HF (2 points), and hyperthyroidism (1 point). These risk factors were determined based on a combination of medical record review and interview at baseline visits. Of note, since there were no data on “coronary artery disease” in the TOPCAT trial dataset, we modified the coronary artery disease criterion using a history of myocardial infarction (MI, as 1 point). Besides, since our studied population was HFpEF patients with merely subtle abnormalities in systolic function [12], the item “systolic HF” in the C_2_HEST score received no point in our current study. The included patients were classified into three risk strata according to C_2_HEST score: the low-risk of 0 to 1 point, the medium-risk of 2 to 3 points, and the high-risk of ≥ 4 points [7].

### Follow-up and outcome determination

Participants were followed up every 4 months during their first year on the study, and every 6 months thereafter, to monitor the events. The first onset of AF during follow-up was the observed endpoint, which was defined as an irregular rhythm with no discernible P-waves confirmed by a physician as AF after ECGs or rhythm strips were adjudicated by a critical event committee. Besides, we also studied the outcomes of stroke, all-cause death, cardiovascular death, any hospitalization, and HF hospitalization, definitions of which were previously described [11]. Data on participants who did not have an event of time-to-event outcomes were censored at the date of last available follow-up information for clinical events.

### Statistical analysis

Continuous variables were presented as mean ± standard deviation and compared using the unpaired Student’s *t* tests (following normal distribution). Categorical variables were presented as proportions and compared using the chi-square test or Fisher’s test as appropriate. Kaplan-Meier curves with the log-rank test were plotted to display the differences of AF, stroke, all-cause death, cardiovascular death, any hospitalization, and HF hospitalization according to the risk strata of the C_2_HEST score, and death was censored in non-death outcomes. A Cox proportional hazard model was used to explore the association of the C_2_HEST score, its components, and its risk strata with all-cause death, and competing risk regression models for cumulative incidence were used when outcomes were incident AF, stroke, cardiovascular death, any hospitalization, and HF hospitalization, and results were reported with hazard ratios (HRs) and confidence intervals (CIs). Death was the competing risk in models concerning incident AF, stroke, any hospitalization, and HF hospitalization, and non-cardiovascular death was the competing risk for cardiovascular death. The adjusted model included various variables, including gender, treatment arm, diabetes mellitus, smoke or ever smoke, body mass index, heart rate, diastolic blood pressure, and estimated glomerular filtration rate. The score was evaluated through the time-dependent (at follow-up at 5 years) area under the curve (AUC) for the receiver operating characteristic (ROC) curve for predicting incident AF and other outcomes. Time-dependent ROC was performed following the methods introduced by Blanche et al. [13]. In brief, it defines cases and controls by subjects with events and censored, and then uses the inverse probability of censoring weighting (IPCW) approach to calculated time-dependent AUC. Blanche et al. also introduced two ways to define controls: (i) a control is defined as a subject *i* that is free of any event, and (ii) a control is defined as a subject *i* that is not a case, and we used definition (i) in the present study.

In a sensitivity analysis, we repeated the above-mentioned analyses by replacing “systolic HF” with “HF.” As reported previously [14, 15], we also used “thyroid disease” to replace “hyperthyroidism” for another sensitivity analysis. In subgroup analysis, we divided subgroups by region of participants (the Americas versus Russia/Georgia) because of concerns about the quality of enrollment as previously reported [16]. Besides, subgroup analyses divided by gender (males versus females) and treatment arm (spironolactone versus placebo) were also performed. All statistical analyses were performed with R version 4.0 (with packages tableone, survival, cmprsk, and timeROC). A two-tailed *P* value of < 0.05 was considered statistically significant.

## Results

### Baseline patient characteristics

Baseline patient characteristics were summarized in Table 1. The low-risk C_2_HEST score stratum was the youngest, with the lowest prevalence of males, and had the highest mean heart rate, diastolic blood pressure, body mass index, and estimated glomerular filtration rate. These patients in the low-risk stratum had the lowest prevalence of MI, stroke, COPD, hypertension, dyslipidemia, and peripheral artery disease history. Baseline echocardiographic characteristics were summarized in Additional file 1: Table S1, but only a small proportion of enrolled patients had echocardiography data available. Patients in the high-risk stratum had the biggest mean maximal left atrial anterior-posterior diameters.

**Table 1:** Baseline characteristics of HFpEF patients stratified according to the C_2_HEST score risk strata

Variables	Total population (*n* = 2202)	Low risk (*n* = 1116)	Medium risk (*n* = 913)	High risk (*n* = 173)	*P* value
Treatment arm (spirolactone)	1098, 49.9%	543, 48.7%	465, 50.9%	90, 50.2%	0.499
Demographic					
Age (years)	67.01 ± 9.44	63.03 ± 6.72	69.45 ± 10.02	79.77 ± 4.01	< 0.001
Age ≥ 75 years	514, 23.3%	0, 0.0%	341, 37.3%	173, 100.0%	< 0.001
Male gender	997, 45.3%	449, 40.2%	455, 49.8%	93, 53.8%	< 0.001
White race	1923, 87.3%	965, 86.5%	811, 88.8%	147, 85.0%	0.177
Current smoking	280, 12.7%	136, 12.2%	138, 15.1%	6, 3.5%	< 0.001
Ever smoking	732, 33.2%	301, 27.0%	346, 37.9%	85, 49.1%	< 0.001
Alcohol drinking	443, 20.1%	228, 20.4%	176, 19.3%	39, 22.7%	0.556
Physical examination					
Heart rate (bpm)	68.61 ± 10.14	69.27 ± 10.21	68.02 ± 10.06	67.49 ± 9.83	0.007
SBP (mmHg)	130.61 ± 13.93	131.15 ± 13.88	130.07 ± 13.89	130.01 ± 14.45	0.187
DBP (mmHg)	76.70 ± 10.68	78.51 ± 10.40	75.51 ± 10.52	71.27 ± 10.61	< 0.001
BMI (kg/m^2^)	32.05 ± 7.18	32.64 ± 7.58	31.66 ± 6.90	30.32 ± 5.48	< 0.001
NYHA class (III or IV)	650, 29.5%	262, 23.5%	319, 34.9%	69, 39.9%	< 0.001
eGFR [mL/(min*1.73m^2^)]	69.19 ± 20.80	71.62 ± 21.20	67.91 ± 20.00	60.31 ± 19.40	< 0.001
Medical history					
Previous MI	634, 28.8%	29, 2.6%	477, 52.2%	128, 74.0%	< 0.001
Previous stroke	146, 6.6%	57, 5.1%	65, 7.1%	24, 13.9%	< 0.001
COPD	231, 10.5%	8, 0.7%	167, 18.3%	56, 32.4%	< 0.001
Asthma	145, 6.6%	62, 5.6%	66, 7.2%	17, 9.8%	0.064
Hypertension	2024, 91.9%	996, 89.2%	855, 93.6%	173, 100.0%	< 0.001
Dyslipidemia	1297, 58.9%	559, 50.1%	606, 66.4%	132, 76.3%	< 0.001
Thyroid disease	274, 12.4%	123, 11.0%	122, 13.4%	29, 16.8%	0.057
Diabetes mellitus	752, 34.2%	368, 33.0%	314, 34.4%	70, 40.5%	0.151
Peripheral artery disease	217, 9.9%	66, 5.9%	126, 13.8%	25, 14.5%	< 0.001
Medications					
ACEIs or ARBs	1880, 85.4%	978, 87.7%	771, 84.4%	131, 75.7%	< 0.001
Beta blockers	1728, 78.5%	860, 77.1%	733, 80.3%	135, 78.0%	0.225
Diuretics	1728, 78.5%	901, 80.8%	679, 74.4%	148, 85.5%	< 0.001
CCBs	875, 39.7%	464, 41.6%	329, 36.0%	82, 47.4%	0.004
Statins	1143, 51.9%	479, 43.0%	549, 60.1%	115, 66.5%	< 0.001
Aspirin	1622, 73.7%	776, 69.6%	703, 77.0%	143, 82.7%	< 0.001
Warfarin	88, 4.0%	33, 3.0%	44, 4.8%	11, 6.4%	0.027

Values are represented as *n,* % or mean ± SD, as appropriate

*AF* atrial fibrillation, *SBP* systolic blood pressure, *DBP* diastolic blood pressure, *BMI* body mass index, *NYHA* New York Heart Association, *eGFR* estimated glomerular filtration rate, *CAD* coronary artery disease, *COPD* chronic obstructive pulmonary disease, *ACEI* angiotensin-converting enzyme inhibitor, *ARB* angiotensin receptor blocker, *CCB* calcium channel blocker

### Association of the C_2_HEST score with AF risk

Among 2202 patients included in our study, 130 (5.9%) incident AF events were recorded during a median follow-up time of 3.07 years. The incidence rates of AF across the C_2_HEST scores were presented in Additional file 1: Table S2. Overall, the average incidence rate of AF was 1.79 per 100 person-years in HFpEF patients. Baseline characteristics of HFpEF patients with or without incident AF were presented in Additional file 1: Table S3.

**Fig. 1:**
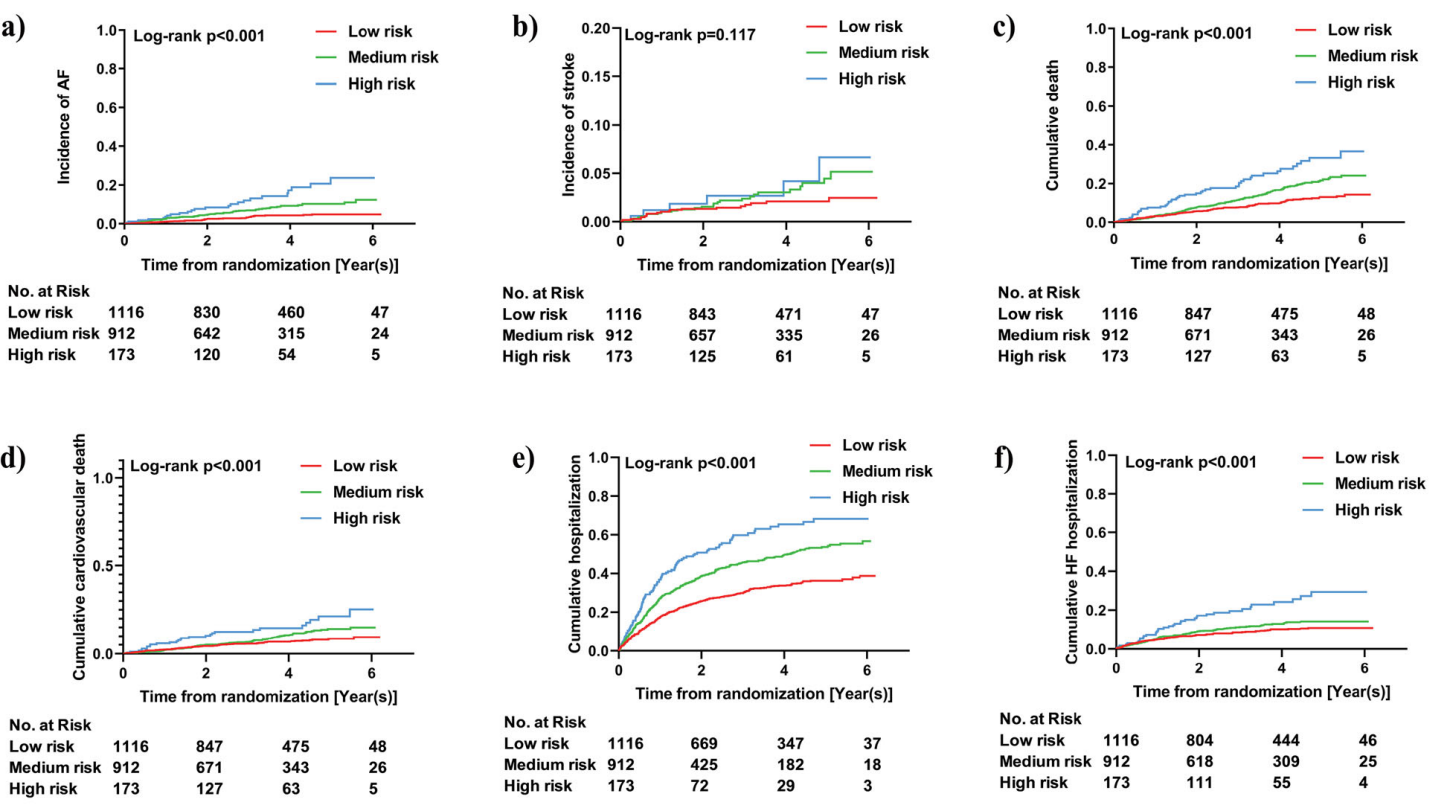
Kaplan–Meier curves for cumulative events of **a** atrial fibrillation, **b** stroke, **c** all-cause death, **d** cardiovascular death, **e** any hospitalization, and **f** heart failure hospitalization during follow-up according to the C_2_HEST risk strata

The associations of individual components in the C_2_HEST score with incident AF are presented in Additional file 1: Table S4. In the competing risk regression models, age ≥ 75 years old (HR 3.21, 95% CI 2.28–4.53) and hyperthyroidism (HR 5.31, 95% CI 2.16–13.10) were independently associated with an increased risk of incident AF. When analyzed as a continuous variable, a 1-point increase in the C_2_HEST score was associated with a 50% increased risk of incident AF (HR 1.50, 95% CI 1.29–1.75; Table 2). When patients were divided into three risk strata, the Kaplan-Meier curves showed a graded increased risk for incident AF (log-rank *P* < 0.001, Fig. 1). Compared with patients in low-risk stratum, those in medium-risk stratum (HR 2.00, 95% CI 1.34–2.99) or high-risk stratum (HR 3.32, 95% CI 1.93–5.71) showed increased risks of incident AF (Table 2).

**Table 2:** Association of the C_2_HEST score with AF and other outcomes in HFpEF patients

Outcomes	Events	Person-years	Incidence rate, per 100 person-years	Unadjusted			Adjusted*		
				HR	95% CI	*P* value	HR	95% CI	*P* value
AF									
Overall**	130, 5.9%	7264	1.79	1.59	1.38–1.83	< 0.001	1.50	1.29–1.75	< 0.001
Risk strata									
Low risk	39, 3.5%	3832	1.02	Ref.			Ref.		
Medium risk	67, 7.3%	2902	2.31	2.18	1.47–3.24	< 0.001	2.00	1.34–2.99	< 0.001
High risk	24, 13.9%	529	4.54	4.04	2.44–6.71	< 0.001	3.32	1.93–5.71	< 0.001
Stroke									
Overall**	52, 2.4%	7396	0.70	1.26	1.01–1.57	0.038	1.24	1.00–1.54	0.054
Risk strata									
Low risk	20, 1.8%	3870	0.52	Ref.			Ref.		
Medium risk	26, 2.8%	2977	0.87	1.64	0.91–2.95	0.097	1.59	0.90–2.79	0.110
High risk	6, 3.5%	550	1.09	1.92	0.77–4.77	0.160	1.77	0.71–4.39	0.220
All-cause death									
Overall**	285, 12.9%	7485	3.81	1.35	1.22–1.49	< 0.001	1.20	1.08–1.33	< 0.001
Risk strata									
Low risk	106, 9.5%	3899	2.72	Ref.			Ref.		
Medium risk	135, 14.8%	3025	4.46	1.65	1.28–2.12	< 0.001	1.39	1.07–1.81	0.012
High risk	27, 15.6%	561	4.81	2.90	2.04–4.13	< 0.001	1.98	1.37–2.86	< 0.001
Cardiovascular death									
Overall**	181, 8.2%	7485	2.42	1.25	1.09–1.43	0.001	1.15	1.00–1.33	0.058
Risk strata									
Low risk	72, 6.5%	3899	1.85	Ref.			Ref.		
Medium risk	82, 9.0%	3025	2.71	1.44	1.05–1.97	0.025	1.26	0.91–1.75	0.170
High risk	27, 15.6%	561	4.81	2.48	1.59–3.87	< 0.001	1.89	1.16–3.09	0.011
Any hospitalization									
Overall**	865, 39.3%	5580	15.50	1.32	1.24–1.40	< 0.001	1.22	1.14–1.29	< 0.001
Risk strata									
Low risk	349, 31.3%	3159	11.05	Ref.			Ref.		
Medium risk	412, 45.1%	2075	19.86	1.65	1.43–1.90	< 0.001	1.50	1.30–1.73	< 0.001
High risk	104, 60.1%	346	30.06	2.41	1.94–3.00	< 0.001	1.87	1.47–2.37	< 0.001
HF hospitalization									
Overall**	235, 10.7%	7072	3.32	1.29	1.16–1.45	< 0.001	1.14	1.01–1.29	0.036
Risk strata									
Low risk	98, 8.8%	3732	2.63	Ref.			Ref.		
Medium risk	100, 11.0%	2834	3.53	1.27	0.96–1.68	0.090	1.10	0.83–1.46	0.520
High risk	37, 21.4%	506	7.31	2.50	1.72–3.64	< 0.001	1.71	1.10–2.64	0.016

*AF* atrial fibrillation, *HR* hazard ratio, *CI* confidence interval

*Variables for adjustment: gender, treatment arm, diabetes mellitus, smoke or ever smoke, body mass index, heart rate, diastolic blood pressure, estimated glomerular filtration rate

**C_2_HEST score was included as a continuous variable

### Association of the C_2_HEST score with other outcomes

Among the studied patients, the average incidence rates of stroke, all-cause death, cardiovascular death, any hospitalization, and HF hospitalization were 0.70, 3.81, 2.42, 15.50, and 3.32 per 100 person-years in HFpEF patients, respectively. The associations of individual components in the C_2_HEST score with outcomes were presented in Additional file 1: Table S4. In Cox proportional hazard and competing risk regression models, previous MI history was significantly associated with higher risks of all-cause death (HR 1.32, 95% CI 1.03–1.68), cardiovascular death (HR 1.39, 95% CI 1.03–1.88), and any hospitalization (HR 1.22, 95% CI 1.06–1.40); COPD history was significantly associated with higher risks of any hospitalization (HR 2.09, 95% CI 1.75–2.50) and HF hospitalization (HR 2.26, 95% CI 1.64–3.11); age ≥ 75 years old was significantly associated with higher risks of all-cause death (HR 1.99, 95% CI 1.56–2.53), cardiovascular death (HR 1.50, 95% CI 1.09–2.06), any hospitalization (HR 1.60, 95% CI 1.38–1.85), and HF hospitalization (HR 1.63, 95% CI 1.24–2.13); thyroid disease history was significantly associated with higher risks of any hospitalization (HR 1.55, 95% CI 1.30–1.85) and HF hospitalization (HR 1.53, 95% CI 1.09–2.14); and hyperthyroidism was significantly associated with higher risk of HF hospitalization (HR 4.08, 95% CI 2.01–8.30).

When analyzed as a continuous variable, per 1-point increase in the C_2_HEST score was associated with increased risks of all-cause death (HR 1.20, 95% CI 1.08–1.33), cardiovascular death (HR 1.15, 95% CI 1.00–1.33), any hospitalization (HR 1.22, 95% CI 1.14–1.29), and HF hospitalization (HR 1.14, 95% CI 1.01–1.29), but not stroke (HR 1.24, 95% CI 1.00–1.54) (Table 2). The cumulative incidences of these outcomes in different risk strata were shown in Fig. 1, and the differences in all-cause death, cardiovascular death, any hospitalization, and HF hospitalization risks among different risk strata were of statistical significance (all *P* < 0.001). In Cox proportional hazard and competing risk regression models, compared with patients in low-risk stratum, those in medium-risk stratum had greater risks of all-cause death (HR 1.39, 95% CI 1.07–1.81) and any hospitalization (HR 1.50, 95% CI 1.30–1.73), but not stroke, cardiovascular death, or HF hospitalization, whereas those in high-risk stratum showed increased risks of all-cause death (HR 1.98, 95% CI 1.37–2.86), cardiovascular death (HR 1.89, 95% CI 1.16–3.09), any hospitalization (HR 1.87, 95% CI 1.47–2.37), and HF hospitalization (HR 1.71, 95% CI 1.10–2.64), except for stroke (Table 2).

### Discriminatory performance of the C_2_HEST score

**Fig. 2:**
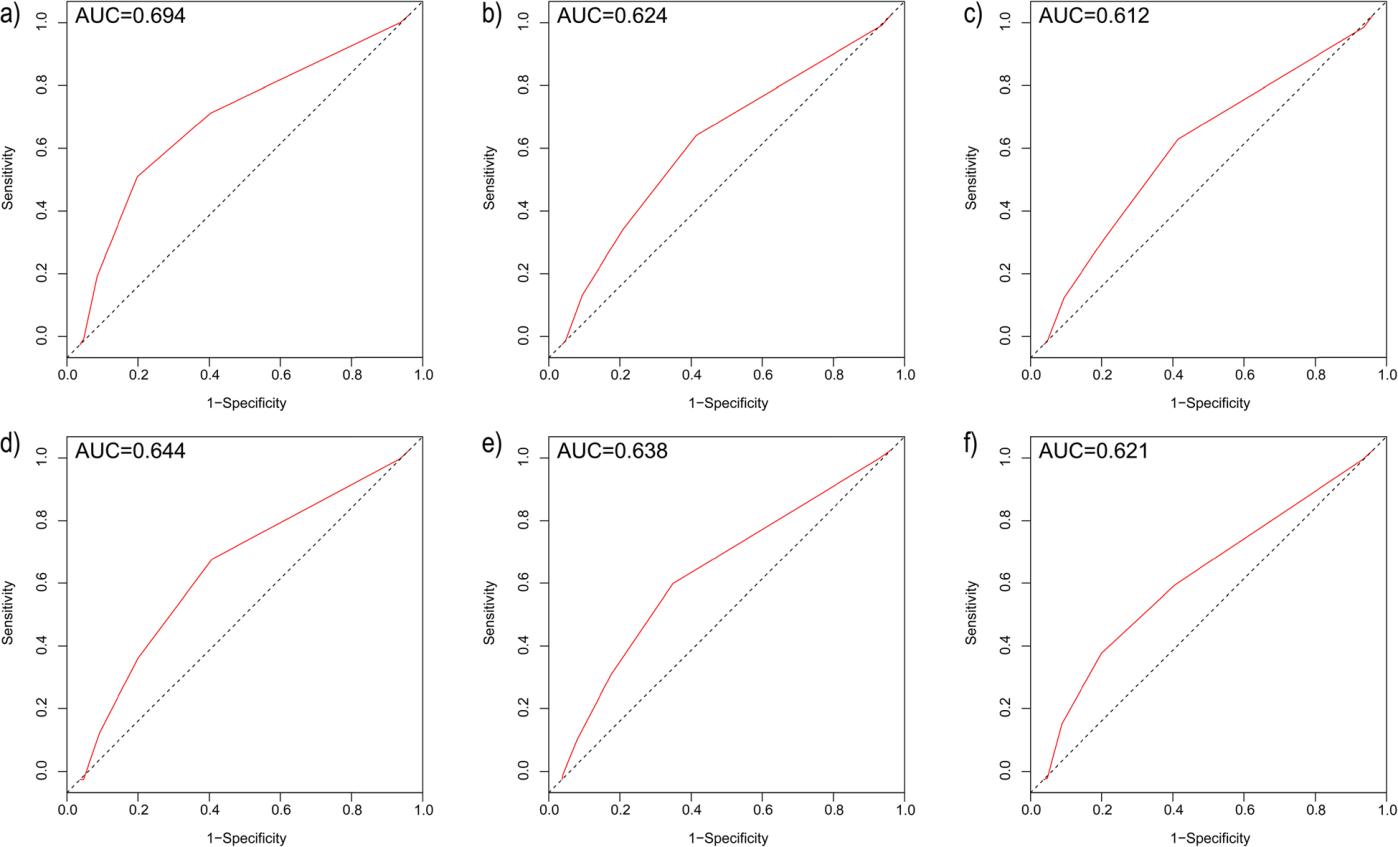
Receiver operating characteristic (ROC) curves for the C_2_HEST score in predicting **a** atrial fibrillation, **b** all-cause death, **c** cardiovascular death, **d** stroke, **e** any hospitalization, and **f** heart failure hospitalization during follow-up

As shown in Fig. 2, the time-dependent AUC for the C_2_HEST score in predicting incident AF (0.694 [95% CI 0.640–0.748]) was higher than other studied outcomes including stroke (0.644 [95% CI 0.565–0.727], all-cause death (0.624 [95% CI 0.583–0.664]), cardiovascular death (0.612 [95% CI 0.564–0.660]), any hospitalization (0.638 [95% CI 0.606–0.670]), or HF hospitalization (0.621 [95% CI 0.577–0.665]).

### Sensitivity analysis

The results of the sensitivity analysis were summarized in Additional file 1: Table S5. When we used “HF” as a scoring item instead of “systolic HF” or replacing “hyperthyroidism” with “thyroid disease,” the results were similar with primary analyses as mentioned above. Performances of the C_2_HEST score on incident AF and other outcomes were similar with primary analyses (Additional file 1: Fig. S1–S2).

### Subgroup analyses

The results of subgroup analyses were summarized in Additional file 1: Table S6. In the subgroup analysis based on region, we observed no significant difference between participants from the Americas and Russia/Georgia regarding the outcomes of AF, stroke, and HF hospitalization (all *P* for interaction > 0.05). The C_2_HEST score was shown to be predictive for all-cause death and cardiovascular death only in participants from Russia/Georgia, but not in those from the Americas. The predictive value of the C_2_HEST score was significantly greater in those from Russia/Georgia than in those from the Americas (all *P*_interaction_ < 0.05). For all outcomes of interest, there were no significant differences in subgroup analyses based on sex (males versus females) or treatment arm (spironolactone versus placebo) (all *P*_interaction_ > 0.05).

## Discussion

Based on the data of the TOPCAT trial, this was the first study designed to assess the predictive performances of the C_2_HEST score for predicting incident AF as well as other trial adjudicated outcomes including stroke, death, and hospitalization in symptomatic HFpEF patients. Our results indicate that the C_2_HEST score analyzed as a continuous variable or a categorical variable was associated with risks of AF, all-cause death, cardiovascular death, any hospitalization, and HF hospitalization, and had moderately good abilities for predicting the development of these adverse outcomes. In addition, the C_2_HEST score was not predictive of stroke risk in HFpEF patients. However, the predictive value of the C_2_HEST score for non-AF outcomes might be altered in patients from different regions.

In addition to HF being associated with a higher incidence rate of AF [17, 18], patients with HFpEF are at a greater risk for AF than those with HFrEF [19]. Undiagnosed AF is common in HF patients and older populations [20]. Moreover, AF impairs cardiac function [21] and increases the risk of thromboembolic complications among HF patients. Therefore, a comprehensive screening program for AF might be necessary in this population. In the community, screening for AF in the elderly has been recommended by pulse-taking [22] or ECG rhythm strip [21]. It is uncertain whether systematic screening of all patients or more opportunistic screening focused on high-risk patients would be best, especially where health-care resources and monitoring equipment availability are limited. Currently, there are still no specific screening strategies or validated risk scoring systems, for incident AF in patients with HFpEF.

Indeed, little is known about the clinical risk stratification to help stratify HFpEF patients for incident AF. The high risk of AF-related adverse outcomes in HFpEF patients justifies the need for an aggressive screening method so that management, such as oral anticoagulation treatment can be initiated without delay. Patients with HF and AF should generally be anticoagulated after balancing the benefits of stroke reduction and risk of bleeding [23], but there are limitations, such as drug interactions with vitamin K antagonists from the polypharmacy in HF management [24]. Early identification of AF in HFpEF patients through a risk stratification tool may help establish a more proper treatment plan.

Several established risk models have specifically been derived for predicting incident AF in the general population, such as the Framingham risk score [25], the Atherosclerosis Risk In Communities Study (ARIC) score [26], HATCH [27], and CHARGE-AF (Cohorts for Heart and Aging Research in Genomic Epidemiology) [28]. The CHADS_2_ and CHA_2_DS_2_-VASc scores [29] have also been used to predict incident AF, although these scores were originally derived and designed for the stroke risk stratification in AF patients. Although all these risk scores have fair discrimination for predicting AF, some are relatively complex due to the requirements of several detailed instrumental and laboratory parameters that may not be immediately available, limiting the use of these scores in everyday clinical practice. In addition, the risk score developed by Kokubo et al. [30] is complex (including > 16 points), and requires data of cardiac murmur, a subjective variable based on physician auscultation. Also, a recent joint consensus document from EHRA, HRS, APHRS, and LAHRS appealed that risk scores should be used for the reason they were proposed and not for reasons they were not designed for or validated [31]. Although other newly scoring systems have been proposed by including genetic risk factors [32, 33] or using a random forest model methodology [34], they could not be used immediately in an opportunistic screening setup.

Concerning the simplest score, the C_2_HEST score, which has been validated in both Asians [7] and non-Asians [8], and shows good discrimination for predicting AF in the post-ischemic stroke patients [9]. The current study further demonstrates that the C_2_HEST score could become a simple practical tool based on clinical risk factors to stratify the risk of incident AF in HFpEF patients. The C_2_HEST score has a moderate predictive ability for AF in patients with HFpEF. Considering that HF patients may be mixed with other populations for risk stratification in practical situations, yet HFpEF is an identified risk factor for AF [17–19], it may be reasonable to count HF into the scoring scheme. When we used “thyroid disease” in scoring rather than “hyperthyroidism”, the HRs of medium-risk and high-risk group both decreased, suggesting that hypothyroidism might be a less definitive risk for incident AF [35, 36] in patients with HFpEF.

Individual risk stratification for incident AF is vital for decision-making of AF screening strategies and early primary prevention measures that may mitigate adverse outcomes [37]. Patients categorized as a high risk for AF could be considered for more intensive heart-rate monitoring for opportunistic AF, such as more frequent ECG examination, 1 to 2 weeks of Holter monitoring at fixed period or an implantable loop recorder employing and so on. Smartwatches could help discover asymptomatic AF, but these wearable devices which were currently designed for consumer use rather than disease screening mainly aim at minimizing false-positive findings [38, 39]. The benefits of diagnosing and treating asymptomatic AF is reflected by the complications and poor prognosis of such individuals detected by screening [40], as well as the beneficial effect of anticoagulant therapy in such patients [41]. In addition to early detection of AF, prevention strategies are also available for those high-risk patients, such as the use of angiotensin-converting enzyme inhibitors and angiotensin receptor blockers, which reduce the risk of incident AF [42, 43].

To our knowledge, the present study is the first demonstration that the C_2_HEST score could predict adverse outcomes including death and hospitalization among patients with HFpEF. Prior studies have found modest predictive values for the CHADS_2_ or CHA_2_DS_2_-VASc scores in predicting adverse outcomes among HF patients from different settings, such as hospitalized patients for new-onset or prevalent HF, discharged HF patients, patients candidate to cardiac resynchronization therapy, and acute decompensated HF patients. Our prior study found that the CHA_2_DS_2_-VASc score could predict the risks of clinical outcomes in HFpEF patients [44]. While all these scores were originally derived and designed for use in AF patients, and not a prognostic assessment in HFpEF, their simplicity and common use in AF allows the possibility of quick risk assessments in busy clinical settings.

### Limitations

We acknowledged several limitations in this study. First, the regional variation of the TOPCAT population might affect results as shown in subgroup analysis, along with our slight modification of the C_2_HEST score criteria to use what was available in the TOPCAT dataset (e.g., using MI to stand for coronary artery disease). Second, our results were based on the data of a retrospective analysis of the TOPCAT trial and it is possible that healthier patients were selected and the unmeasured confounders were not found, which might influence the validity of our findings. Third, incident AF as the outcome event was collected in the follow-up every 4 or 6 months. Coupled with some missed visits, there could be a certain degree of underestimation of incident AF. In addition, some participants with paroxysmal AF might not be aware of it and not recorded when they received ECG examination at baseline, and therefore, the follow-up outcome in our study might be recurrent AF.

## Conclusions

The C_2_HEST score could predict the risk of incident AF as well as death and hospitalization with moderately good predictive ability in patients with HFpEF. The simplicity of the C_2_HEST score may allow the possibility of quick risk assessments in busy clinical settings.

## Supplementary Information

**Additional file 1: Table S1.** Baseline echocardiographic characteristics of HFpEF patients stratified according to the C2HEST score risk strata. **Table S2.** The C2HEST score and the risk of incident AF in the competing risk regression model. **Table S3.** Baseline characteristics of patients with and without incident AF during follow-up. **Table S4.** Components of C2HEST score and the risk of outcomes in univariate Cox proportional hazard model (all-cause death) and competing risk regression model (other outcomes). **Table S5.** Sensitivity analyses of C2HEST score risk strata and the risk of AF and other outcomes. **Table S6.** Subgroup analysis (C2HEST score was included as continuous variable). **Fig. S1.** Receiver operating characteristic (ROC) curves for the C2HEST score (with “HF” as a scoring item instead of “systolic HF”) in predicting a) atrial fibrillation, b) all-cause death, c) cardiovascular death, d) stroke, e) any hospitalization and f) heart failure hospitalization during follow-up. **Fig. S2.** Receiver operating characteristic (ROC) curves for the C2HEST score (with “hyperthyroidism” replaced by “thyroid disease”) in predicting a) atrial fibrillation, b) all-cause death, c) cardiovascular death, d) stroke, e) any hospitalization and f) heart failure hospitalization during follow-up.

## Data Availability

The dataset of the TOPCAT trial is available via reasonable request to the National Heart, Lung and Blood Institution.

## Abbreviations

HF: Heart failure
HFpEF: Heart failure with preserved ejection fraction
AF: Atrial fibrillation
LVEF: Left ventricular ejection fraction
ECG: Electrocardiogram
COPD: Chronic obstructive pulmonary disease
MI: Myocardial infarction
HR: Hazard ratio
CI: Confidence interval
AUC: Area under the curve
ROC: Receiver operating characteristic
ARIC: Atherosclerosis Risk In Communities
