# Supplementary material for: C2HEST score predicts clinical outcomes in heart failure with preserved ejection fraction: a secondary analysis of the TOPCAT trial
Source: BMC Med. 2021 Feb 18;19:44. doi: 10.1186/s12916-021-01921-w (PMC7890599; doi:10.1186/s12916-021-01921-w)
Supplement: Supplementary file 1 — Additional file 1: Table S1. Baseline echocardiographic characteristics of HFpEF patients stratified according to the C2HEST score risk strata. Table S2. The C2HEST score and the risk of incident AF in the competing risk regression model. Table S3. Baseline characteristics of patients with and without incident AF during follow-up. Table S4. Components of C2HEST score and the risk of outcomes in univariate Cox proportional hazard model (all-cause death) and competing risk regression model (other outcomes). Table S5. Sensitivity analyses of C2HEST score risk strata and the risk of AF and other outcomes. Table S6. Subgroup analysis (C2HEST score was included as continuous variable). Fig. S1. Receiver operating characteristic (ROC) curves for the C2HEST score (with “HF” as a scoring item instead of “systolic HF”) in predicting a) atrial fibrillation, b) all-cause death, c) cardiovascular death, d) stroke, e) any hospitalization and f) heart failure hospitalization during follow-up. Fig. S2. Receiver operating characteristic (ROC) curves for the C2HEST score (with “hyperthyroidism” replaced by “thyroid disease”) in predicting a) atrial fibrillation, b) all-cause death, c) cardiovascular death, d) stroke, e) any hospitalization and f) heart failure hospitalization during follow-up. [file 12916_2021_1921_MOESM1_ESM.docx]

**Table S1. Baseline echocardiographic characteristics of HFpEF patients stratified according to the C2HEST score risk strata.**

| Variables | Low risk | Medium risk | High risk | P value |
| --- | --- | --- | --- | --- |
| Eccentric mitral regurgitation (n=341) | 1, 0.7% | 2, 1.3% | 0, 0% | 0.685 |
| Moderate or greater mitral regurgitation (n=341) | 11, 7.3% | 18, 11.8% | 6, 15.4% | 0.232 |
| Interventricular septum thickness (n=526) | 1.18±0.21 | 1.20±0.20 | 1.22±0.18 | 0.555 |
| Posterior wall thickness (n=526) | 1.15±0.20 | 1.15±0.19 | 1.18±0.18 | 0.588 |
| End-diastolic left ventricular diameter (n=526) | 4.78±0.60 | 4.80±0.55 | 4.87±0.58 | 0.503 |
| Maximal left atrial anterior-posterior diameter (n=526) | 4.04±0.60 | 4.08±0.53 | 4.29±0.56 | 0.012 |
| Lateral early diastolic myocardial velocity (n=307) | 7.93±2.89 | 7.06±2.69 | 7.64±2.51 | 0.034 |
| Lateral late diastolic myocardial velocity (n=302) | 8.99±3.88 | 8.80±3.24 | 9.25±3.44 | 0.770 |
| Mitral regurgitation jet area (n=366) | 1.04±1.36 | 1.27±1.97 | 1.66±2.18 | 0.104 |
| E wave deceleration time (n=425) | 214.87±70.39 | 216.60±59.65 | 190.95±52.03 | 0.033 |
| Peak tricuspid regurgitation velocity (n=203) | 277.19±47.57 | 272.26±44.40 | 283.64±49.92 | 0.533 |
| E/A ratio (n=418) | 1.18±0.54 | 1.09±0.53 | 1.08±0.69 | 0.237 |
| End-systolic left ventricular diameter (n=526) | 3.34±0.54 | 3.35±0.49 | 3.45±0.50 | 0.343 |
| Septal early diastolic myocardial velocity (n=294) | 6.03±1.91 | 5.55±2.49 | 5.42±1.95 | 0.123 |
| Septal late diastolic myocardial velocity (n=288) | 7.84±2.49 | 7.78±2.71 | 7.95±2.44 | 0.941 |
| RV end diastolic area (n=396) | 20.10±6.50 | 19.05±4.60 | 19.75±6.58 | 0.232 |
| RV end systolic area (n=396) | 10.07±3.83 | 9.72±3.20 | 9.70±3.29 | 0.610 |
| Lateral systolic myocardial velocity (n=306) | 7.31±2.50 | 6.66±1.94 | 6.76±2.45 | 0.055 |
| Septal systolic myocardial velocity (n=291) | 6.27±1.96 | 6.13±2.03 | 5.96±1.62 | 0.671 |
| Left atrial area (n=478) | 18.02±4.58 | 17.65±4.30 | 18.22±4.13 | 0.571 |
| Mean LV wall thickness (n=526) | 1.17±0.21 | 1.17±0.19 | 1.20±0.18 | 0.583 |
| LV Mass (n=526) | 215.24±72.79 | 217.77±67.25 | 227.69±63.42 | 0.481 |
| Ejection Fraction (n=565) | 59.76±7.95 | 59.94±7.48 | 59.76±8.40 | 0.963 |
| E/Em septal ratio (n=290) | 15.71±6.74 | 15.38±6.18 | 16.77±8.94 | 0.557 |
| RV fractional area change (n=396) | 0.50±0.08 | 0.49±0.08 | 0.51±0.06 | 0.571 |
| E/Em lateral ratio (n=304) | 11.92±5.49 | 12.11±4.97 | 11.54±4.95 | 0.837 |
| Teicholtz end-diastolic volume (n=565) | 108.06±32.40 | 108.25±30.27 | 113.69±30.86 | 0.454 |
| Teicholtz end-systolic volume (n=565) | 46.92±19.50 | 46.46±17.85 | 50.69±17.67 | 0.301 |
| Teicholtz Ejection fraction (n=560) | 56.70±8.00 | 55.86±9.03 | 55.98±5.59 | 0.502 |
| LV relative wall thickness (n=526) | 0.49±0.11 | 0.48±0.10 | 0.49±0.10 | 0.892 |
| MR jet area-to-left atrial area ratio (n=341) | 0.06±0.07 | 0.06±0.09 | 0.09±0.10 | 0.180 |

**Table S2. The C_2_HEST score and the risk of incident AF in the competing risk regression model.**

| **Score** | **Events** | **Person-years** | **Incidence rate,**  **per 100 person-years** | **Unadjusted** | | |  | **Adjusted*** | | |
| --- | --- | --- | --- | --- | --- | --- | --- | --- | --- | --- |
|  |  |  |  | **HR** | **95%CI** | **P value** |  | **HR** | **95%CI** | **P value** |
| 0 | 4, 4.8% | 272 | 1.47 | Ref. |  |  |  | Ref. |  |  |
| 1 | 35, 3.4% | 3560 | 0.98 | 0.72 | 0.25-2.04 | 0.530 |  | 0.67 | 0.24-1.90 | 0.450 |
| 2 | 26, 4.7% | 1793 | 1.45 | 1.01 | 0.35-2.95 | 0.980 |  | 0.89 | 0.31-2.58 | 0.830 |
| 3 | 41, 11.2% | 1108 | 3.70 | 2.56 | 0.90-7.28 | 0.077 |  | 2.20 | 0.79-6.18 | 0.130 |
| 4 | 22, 14.3% | 467 | 4.71 | 3.10 | 1.05-9.14 | 0.040 |  | 2.55 | 0.87-7.44 | 0.087 |
| 5 | 2, 10.5% | 62 | 3.23 | 2.07 | 0.38-11.17 | 0.400 |  | 1.37 | 0.22-8.43 | 0.740 |
| As continuous variable | 130, 5.9% | 7264 | 1.79 | 1.59 | 1.38-1.83 | <0.001 |  | 1.50 | 1.29-1.75 | <0.001 |

*Variables for adjustment: gender, treatment arm, diabetes mellitus, smoke or ever smoke, body mass index, heart rate, diastolic blood pressure, eGFR.

**Table S3. Baseline characteristics of patients with and without incident AF during follow-up.**

| Variables | With incident AF  (n=130) | Without incident AF  (n=2072) |
| --- | --- | --- |
| Treatment arm (spirolactone) | 64, 49.2% | 1040, 50.2% |
| Demographic |  |  |
| Age (yrs) | 72.38±8.84 | 66.67±9.38 |
| Age ≥75 yrs | 62, 47.7% | 452, 21.8% |
| Male gender | 70, 53.8% | 927, 44.7% |
| White race | 110, 84.6% | 1813, 87.5% |
| Current smoking | 8, 6.2% | 272, 13.1% |
| Ever smoking | 63, 48.5% | 669, 32.3% |
| Alcohol drinking | 21, 16.2% | 422, 20.4% |
| Physical examination |  |  |
| Heart rate (bpm) | 66.35±10.71 | 68.76±10.09 |
| SBP (mmHg) | 130.45±14.62 | 130.62±13.89 |
| DBP (mmHg) | 72.21±11.69 | 76.98±10.55 |
| BMI (kg/m^2^) | 33.27±7.05 | 31.97±7.18 |
| NYHA class (III or IV) | 44, 33.8% | 606, 29.3% |
| eGFR [mL/(min*1.73m^2^)] | 65.83±22.04 | 69.40±20.71 |
| Medical history |  |  |
| Previous MI | 41, 31.5% | 593, 28.6% |
| Stroke | 11, 8.5% | 135, 6.5% |
| COPD | 19, 14.6% | 212, 10.5% |
| Asthma | 9, 6.9% | 136, 6.6% |
| Hypertension | 119, 91.5% | 1905, 91.9% |
| Dyslipidemia | 91, 70.0% | 1206, 58.2% |
| Thyroid disease | 20, 15.4% | 254, 12.3% |
| Diabetes mellitus | 62, 47.7% | 690, 33.3% |
| Peripheral artery disease | 24, 18.5% | 193, 9.3% |
| Medications |  |  |
| ACEIs or ARBs | 104, 80.0% | 1776, 85.8% |
| Beta blockers | 108, 83.1% | 1620, 78.2% |
| Diuretics | 112, 86.2% | 1616, 78.0% |
| CCBs | 57, 43.8% | 818, 39.5% |
| Statins | 85, 65.4% | 1058, 51.1% |
| Aspirin | 102, 78.5% | 1520, 73.4% |
| Warfarin | 8, 6.2% | 80, 3.9% |

AF: atrial fibrillation, SBP: systolic blood pressure, DBP: diastolic blood pressure, BMI: body mass index, NYHA: New York Heart Association, eGFR: estimated glomerular filtration rate, MI: myocardial infarction, COPD: chronic obstructive pulmonary disease, ACEI: angiotensin converting enzyme inhibitor, ARB: angiotensin receptor blocker.

**Table S4. Components of C_2_HEST score and the risk of outcomes in univariate Cox proportional hazard model (all-cause death) and competing risk regression model (other outcomes).**

| Components | Events* | Person-years* | Incidence rate*,  per 100 person-years | Control incidence rate**,  per 100 person-years | HR | 95%CI | P value |
| --- | --- | --- | --- | --- | --- | --- | --- |
| AF |  |  |  |  |  |  |  |
| Previous MI | 41, 6.5% | 2156 | 1.90 | 1.74 | 1.08 | 0.74-1.56 | 0.700 |
| COPD | 19, 8.1% | 694 | 2.74 | 1.69 | 1.53 | 0.94-2.49 | 0.084 |
| Elderly (age≥75) | 62, 12.1% | 1533 | 4.04 | 1.19 | 3.21 | 2.28-4.53 | <0.001 |
| Hypertension | 119, 5.9% | 6680 | 1.78 | 1.88 | 0.97 | 0.52-1.81 | 0.920 |
| Thyroid disease | 20, 7.3% | 867 | 2.31 | 1.72 | 1.30 | 0.81-2.09 | 0.280 |
| Hyperthyroidism | 5, 27.8% | 55 | 9.09 | 1.73 | 5.31 | 2.16-13.10 | <0.001 |
| Stroke |  |  |  |  |  |  |  |
| Previous MI | 18, 2.8% | 2111 | 0.85 | 0.65 | 1.24 | 0.71-2.19 | 0.450 |
| COPD | 8, 3.5% | 695 | 1.15 | 0.66 | 1.66 | 0.78-3.52 | 0.190 |
| Elderly (age≥75) | 15, 2.9% | 1596 | 0.94 | 0.64 | 1.39 | 0.76-2.53 | 0.280 |
| Hypertension | 50, 2.5% | 6795 | 0.74 | 0.33 | 2.25 | 0.55-9.16 | 0.260 |
| Thyroid disease | 7, 2.6% | 891 | 0.79 | 0.69 | 1.11 | 0.50-2.45 | 0.800 |
| Hyperthyroidism | 1, 5.6% | 64 | 1.56 | 0.70 | 2.40 | 0.34-17.20 | 0.380 |
| All-cause death |  |  |  |  |  |  |  |
| Previous MI | 102, 16.1% | 2224 | 4.59 | 3.48 | 1.32 | 1.03-1.68 | 0.026 |
| COPD | 37, 16.0% | 722 | 5.12 | 3.67 | 1.40 | 0.99-1.98 | 0.057 |
| Elderly (age≥75) | 101, 19.6% | 1628 | 6.20 | 3.14 | 1.99 | 1.56-2.53 | <0.001 |
| Hypertension | 259, 12.8% | 6879 | 3.77 | 4.29 | 0.88 | 0.59-1.32 | 0.534 |
| Thyroid disease | 42, 15.3% | 900 | 4.67 | 3.69 | 1.27 | 0.92-1.76 | 0.151 |
| Hyperthyroidism | 2, 11.1% | 64 | 3.13 | 3.81 | 0.83 | 0.21-3.32 | 0.787 |
| Cardiovascular death |  |  |  |  |  |  |  |
| Previous MI | 67, 10.6% | 2224 | 3.01 | 2.17 | 1.39 | 1.03-1.88 | 0.031 |
| COPD | 24, 10.4% | 722 | 3.32 | 2.32 | 1.40 | 0.91-2.15 | 0.130 |
| Elderly (age≥75) | 55, 10.7% | 1628 | 3.38 | 2.15 | 1.50 | 1.09-2.06 | 0.012 |
| Hypertension | 166, 8.2% | 6879 | 2.41 | 2.48 | 0.99 | 0.58-1.68 | 0.960 |
| Thyroid disease | 26, 9.5% | 900 | 2.89 | 2.35 | 1.21 | 0.80-1.84 | 0.360 |
| Hyperthyroidism | 2, 11.1% | 64 | 3.13 | 2.41 | 1.33 | 0.34-5.23 | 0.680 |
| Any hospitalization |  |  |  |  |  |  |  |
| Previous MI | 280, 44.2% | 1538 | 18.21 | 14.47 | 1.22 | 1.06-1.40 | 0.006 |
| COPD | 140, 60.6% | 414 | 33.82 | 14.03 | 2.09 | 1.75-2.50 | <0.001 |
| Elderly (age≥75) | 258, 50.2% | 1103 | 23.39 | 13.56 | 1.60 | 1.38-1.85 | <0.001 |
| Hypertension | 807, 39.9% | 5094 | 15.84 | 11.93 | 1.31 | 1.00-1.72 | 0.049 |
| Thyroid disease | 142, 51.8% | 586 | 24.23 | 14.47 | 1.55 | 1.30-1.85 | <0.001 |
| Hyperthyroidism | 10, 55.6% | 40 | 25.00 | 15.43 | 1.46 | 0.83-2.57 | 0.180 |
| HF hospitalization |  |  |  |  |  |  |  |
| Previous MI | 62, 9.8% | 2118 | 2.93 | 3.49 | 0.85 | 0.63-1.13 | 0.260 |
| COPD | 46, 19.9% | 642 | 7.17 | 2.94 | 2.26 | 1.64-3.11 | <0.001 |
| Elderly (age≥75) | 76, 14.8% | 1503 | 5.06 | 2.86 | 1.63 | 1.24-2.13 | <0.001 |
| Hypertension | 223, 11.0% | 6478 | 3.44 | 2.02 | 1.72 | 0.96-3.08 | 0.069 |
| Thyroid disease | 41, 15.0% | 829 | 4.95 | 3.11 | 1.53 | 1.09-2.14 | 0.014 |
| Hyperthyroidism | 1, 5.6% | 64 | 1.56 | 3.25 | 4.08 | 2.01-8.30 | <0.001 |

AF: atrial fibrillation, HR: hazard ratio, CI: confidence interval, MI: myocardial infarction, COPD: chronic obstructive pulmonary disease.

*Presenting event, person-years and incidence rate of patients **with** risk factors listed in the first column.

**Incidence rate of patients **without** risk factors listed in the first column.

**Table S5. Sensitivity analyses of C_2_HEST score risk strata and the risk of AF and other outcomes.**

| C_2_HEST strata | Events | Person-years | Incidence rate,  per 100 person-years | Unadjusted | | |  | Adjusted* | | |
| --- | --- | --- | --- | --- | --- | --- | --- | --- | --- | --- |
|  |  |  |  | HR | 95%CI | P value |  | HR | 95%CI | P value |
| AF | | | | | | | | | | |
| Replace “systolic heart failure” by “heart failure” | | | | | | | | | | |
| Overall** | 130, 5.9% | 7264 | 1.79 | 1.59 | 1.38-1.83 | <0.001 |  | 1.50 | 1.29-1.75 | <0.001 |
| Risk strata |  |  |  |  |  |  |  |  |  |  |
| Low risk (0-1) | 0, 0% | 0 | - |  |  |  |  |  |  |  |
| Medium risk (2-3) | 39, 3.5% | 3832 | 1.02 | Ref. | | |  | Ref. | | |
| High risk (≥4) | 91, 8.4% | 3431 | 2.65 | 2.48 | 1.71-3.61 | <0.001 |  | 2.20 | 1.50-3.22 | <0.001 |
| Replace “hyperthyroidism” by “thyroid disease” | | | | | | | | | | |
| Overall** | 130, 5.9% | 7264 | 1.79 | 1.53 | 1.34-1.75 | <0.001 |  | 1.44 | 1.25-1.67 | <0.001 |
| Risk strata |  |  |  |  |  |  |  |  |  |  |
| Low risk (0-1) | 35, 3.5% | 3445 | 1.02 | Ref. | | |  | Ref. | | |
| Medium risk (2-3) | 65, 6.6% | 3163 | 2.06 | 1.95 | 1.29-2.94 | 0.002 |  | 1.76 | 1.17-2.64 | 0.007 |
| High risk (≥4) | 30, 14.0% | 655 | 4.58 | 4.09 | 2.52-6.66 | <0.001 |  | 3.25 | 1.93-5.45 | <0.001 |
| Stroke |  |  |  |  |  |  |  |  |  |  |
| Replace “systolic heart failure” by “heart failure” | | | | | | | | | | |
| Overall** | 52, 2.4% | 7396 | 0.70 | 1.26 | 1.01-1.57 | 0.038 |  | 1.25 | 0.97-1.61 | 0.082 |
| Risk strata |  |  |  |  |  |  |  |  |  |  |
| Low risk (0-1) | 0, 0% | 0 | - |  |  |  |  |  |  |  |
| Medium risk (2-3) | 20, 1.8% | 3870 | 0.52 | Ref. |  |  |  | Ref. |  |  |
| High risk (≥4) | 32, 2.9% | 3527 | 0.91 | 1.69 | 0.96-2.96 | 0.068 |  | 1.62 | 0.95-2.76 | 0.079 |
| Replace “hyperthyroidism” by “thyroid disease” | | | | | | | | | | |
| Overall** | 52, 2.4% | 7396 | 0.70 | 1.24 | 1.00-1.54 | 0.054 |  | 1.20 | 0.97-1.48 | 0.097 |
| Risk strata |  |  |  |  |  |  |  |  |  |  |
| Low risk (0-1) | 18, 1.8% | 3483 | 0.52 | Ref. |  |  |  | Ref. |  |  |
| Medium risk (2-3) | 26, 2.6% | 3233 | 0.80 | 1.51 | 0.83-2.76 | 0.180 |  | 1.44 | 0.80-2.59 | 0.230 |
| High risk (≥4) | 8, 3.7% | 680 | 1.18 | 2.08 | 0.90-4.77 | 0.086 |  | 1.83 | 0.80-4.19 | 0.150 |
| All-cause death | | | | | | | | | | |
| Replace “systolic heart failure” by “heart failure” | | | | | | | | | | |
| Overall** | 285, 12.9% | 7485 | 3.81 | 1.35 | 1.22-1.49 | <0.001 |  | 1.20 | 1.08-1.33 | <0.001 |
| Risk strata |  |  |  |  |  |  |  |  |  |  |
| Low risk (0-1) | 0, 0% | 0 | - |  |  |  |  |  |  |  |
| Medium risk (2-3) | 106, 9.5% | 3899 | 2.72 | Ref. |  |  |  | Ref. |  |  |
| High risk (≥4) | 179, 16.5% | 3586 | 4.99 | 1.84 | 1.45-2.34 | <0.001 |  | 1.49 | 1.16-1.91 | 0.002 |
| Replace “hyperthyroidism” by “thyroid disease” | | | | | | | | | | |
| Overall** | 285, 12.9% | 7485 | 3.81 | 1.34 | 1.22-1.47 | <0.001 |  | 1.20 | 1.09-1.33 | <0.001 |
| Risk strata |  |  |  |  |  |  |  |  |  |  |
| Low risk (0-1) | 93, 9.3% | 3509 | 2.65 | Ref. |  |  |  | Ref. |  |  |
| Medium risk (2-3) | 139, 14.1% | 3280 | 4.24 | 1.61 | 1.23-2.09 | <0.001 |  | 1.37 | 1.05-1.79 | 0.021 |
| High risk (≥4) | 53, 24.7% | 697 | 7.60 | 2.89 | 2.06-4.05 | <0.001 |  | 1.98 | 1.39-2.82 | <0.001 |
| Cardiovascular death | | | | | | | | | | |
| Replace “systolic heart failure” by “heart failure” | | | | | | | | | | |
| Overall** | 181, 8.2% | 7485 | 2.42 | 1.25 | 1.09-1.43 | 0.001 |  | 1.15 | 1.00-1.33 | 0.058 |
| Risk strata |  |  |  |  |  |  |  |  |  |  |
| Low risk (0-1) | 0, 0% | 0 | - |  |  |  |  |  |  |  |
| Medium risk (2-3) | 72, 6.5% | 3899 | 1.85 | Ref. |  |  |  | Ref. |  |  |
| High risk (≥4) | 109, 10.0% | 3586 | 3.04 | 1.60 | 1.19-2.16 | 0.002 |  | 1.36 | 0.99-1.87 | 0.058 |
| Replace “hyperthyroidism” by “thyroid disease” | | | | | | | | | | |
| Overall** | 181, 8.2% | 7485 | 2.42 | 1.24 | 1.09-1.41 | <0.001 |  | 1.16 | 1.01-1.33 | 0.042 |
| Risk strata |  |  |  |  |  |  |  |  |  |  |
| Low risk (0-1) | 64, 6.4% | 3509 | 1.82 | Ref. |  |  |  | Ref. |  |  |
| Medium risk (2-3) | 84, 8.5% | 3280 | 2.56 | 1.38 | 0.99-1.91 | 0.055 |  | 1.23 | 0.88-1.73 | 0.230 |
| High risk (≥4) | 33, 15.3% | 697 | 4.73 | 2.48 | 1.63-3.77 | <0.001 |  | 1.95 | 1.22-3.12 | 0.005 |
| Any hospitalization | | | | | | | | | | |
| Replace “systolic heart failure” by “heart failure” | | | | | | | | | | |
| Overall** | 865, 39.3% | 5580 | 15.50 | 1.32 | 1.24-1.40 | <0.001 |  | 1.22 | 1.14-1.29 | <0.001 |
| Risk strata |  |  |  |  |  |  |  |  |  |  |
| Low risk (0-1) | 0, 0% | 0 | - |  |  |  |  |  |  |  |
| Medium risk (2-3) | 349, 31.3% | 3159 | 11.05 | Ref. |  |  |  | Ref. |  |  |
| High risk (≥4) | 516, 47.5% | 2421 | 21.31 | 1.76 | 1.53-2.01 | <0.001 |  | 1.55 | 1.35-1.79 | <0.001 |
| Replace “hyperthyroidism” by “thyroid disease” | | | | | | | | | | |
| Overall** | 865, 39.3% | 5580 | 15.50 | 1.33 | 1.26-1.40 | <0.001 |  | 1.22 | 1.15-1.30 | <0.001 |
| Risk strata |  |  |  |  |  |  |  |  |  |  |
| Low risk (0-1) | 293, 29.3% | 2902 | 10.10 | Ref. |  |  |  | Ref. |  |  |
| Medium risk (2-3) | 442, 44.7% | 2251 | 19.64 | 1.77 | 1.53-2.05 | <0.001 |  | 1.60 | 1.38-1.86 | <0.001 |
| High risk (≥4) | 130, 60.5% | 427 | 30.44 | 2.63 | 2.14-3.23 | <0.001 |  | 1.98 | 1.59-2.48 | <0.001 |
| HF hospitalization | | | | | | | | | | |
| Replace “systolic heart failure” by “heart failure” | | | | | | | | | | |
| Overall** | 235, 10.7% | 7072 | 3.32 | 1.29 | 1.16-1.45 | <0.001 |  | 1.14 | 1.01-1.29 | 0.036 |
| Risk strata |  |  |  |  |  |  |  |  |  |  |
| Low risk (0-1) | 0, 0% | 0 | - |  |  |  |  |  |  |  |
| Medium risk (2-3) | 98, 8.8% | 3732 | 2.63 | Ref. |  |  |  | Ref. |  |  |
| High risk (≥4) | 137, 12.6% | 3340 | 4.10 | 1.47 | 1.13-1.90 | 0.004 |  | 1.26 | 0.91-1.58 | 0.190 |
| Replace “hyperthyroidism” by “thyroid disease” | | | | | | | | | | |
| Overall** | 235, 10.7% | 7072 | 3.32 | 1.29 | 1.16-1.44 | <0.001 |  | 1.13 | 1.00-1.27 | 0.050 |
| Risk strata |  |  |  |  |  |  |  |  |  |  |
| Low risk (0-1) | 84, 8.4% | 3372 | 2.49 | Ref. |  |  |  | Ref. |  |  |
| Medium risk (2-3) | 104, 10.5% | 3077 | 3.38 | 1.28 | 0.96-1.71 | 0.089 |  | 1.09 | 0.81-1.47 | 0.560 |
| High risk (≥4) | 47, 21.9% | 622 | 7.56 | 2.69 | 1.89-3.83 | <0.001 |  | 1.73 | 1.14-2.80 | 0.010 |

AF: atrial fibrillation, HR: hazard ratio, CI: confidence interval.

*Variables for adjustment: gender, treatment arm, diabetes mellitus, smoke or ever smoke, body mass index, heart rate, diastolic blood pressure, estimated glomerular filtration rate.

**C_2_HEST score were included as continuous variable.

**Table S6. Subgroup analysis (C_2_HEST score was included as continuous variable).**

|  |  |  | Unadjusted |  |  |  |  | Adjusted* |  |  |
| --- | --- | --- | --- | --- | --- | --- | --- | --- | --- | --- |
| Subgroups | Number of patients | HR | 95%CI | P value | P for interaction |  | HR | 95%CI | P value | P for interaction |
| **AF** |  |  |  |  |  |  |  |  |  |  |
| Region |  |  |  |  | 0.052 |  |  |  |  | 0.061 |
| Americas | 1005 | 1.30 | 1.12-1.52 | <0.001 |  |  | 1.27 | 1.07-1.49 | 0.006 |  |
| Russia/Georgia | 1197 | 1.85 | 1.35-2.53 | <0.001 |  |  | 1.93 | 1.39-2.68 | <0.001 |  |
| Gender |  |  |  |  | 0.094 |  |  |  |  | 0.060 |
| Male | 997 | 1.41 | 1.15-1.73 | 0.001 |  |  | 1.34 | 1.09-1.64 | 0.006 |  |
| Female | 1205 | 1.79 | 1.49-2.15 | <0.001 |  |  | 1.70 | 1.37-2.11 | <0.001 |  |
| Treatment group |  |  |  |  | 0.640 |  |  |  |  | 0.540 |
| Spironolactone | 1098 | 1.65 | 1.38-1.97 | <0.001 |  |  | 1.59 | 1.30-1.94 | <0.001 |  |
| Placebo | 1104 | 1.54 | 1.24-1.90 | <0.001 |  |  | 1.42 | 1.12-1.78 | 0.003 |  |
| **Stroke** |  |  |  |  |  |  |  |  |  |  |
| Region |  |  |  |  | 0.072 |  |  |  |  | 0.057 |
| Americas | 1005 | 1.25 | 0.99-1.59 | 0.063 |  |  | 1.29 | 1.03-1.62 | 0.025 |  |
| Russia/Georgia | 1197 | 0.79 | 0.52-1.22 | 0.290 |  |  | 0.77 | 0.49-1.24 | 0.280 |  |
| Gender |  |  |  |  | 0.840 |  |  |  |  | 0.730 |
| Male | 997 | 1.31 | 0.93-1.84 | 0.130 |  |  | 1.36 | 0.97-1.92 | 0.079 |  |
| Female | 1205 | 1.25 | 0.94-1.67 | 0.130 |  |  | 1.20 | 0.88-1.62 | 0.250 |  |
| Treatment group |  |  |  |  | 0.870 |  |  |  |  | 0.930 |
| Spironolactone | 1098 | 1.29 | 0.92-1.81 | 0.140 |  |  | 1.25 | 0.89-1.76 | 0.190 |  |
| Placebo | 1104 | 1.24 | 0.93-1.66 | 0.140 |  |  | 1.24 | 0.92-1.65 | 0.150 |  |
| **All-cause death** |  |  |  |  |  |  |  |  |  |  |
| Region |  |  |  |  | 0.027 |  |  |  |  | 0.038 |
| Americas | 1005 | 1.11 | 0.99-1.25 | 0.073 |  |  | 1.03 | 0.91-1.16 | 0.616 |  |
| Russia/Georgia | 1197 | 1.45 | 1.18-1.78 | <0.001 |  |  | 1.42 | 1.14-1.76 | 0.002 |  |
| Gender |  |  |  |  | 0.314 |  |  |  |  | 0.675 |
| Male | 997 | 1.27 | 1.11-1.45 | <0.001 |  |  | 1.19 | 1.03-1.37 | 0.016 |  |
| Female | 1205 | 1.41 | 1.21-1.64 | <0.001 |  |  | 1.18 | 1.00-1.38 | 0.046 |  |
| Treatment group |  |  |  |  | 0.485 |  |  |  |  | 0.447 |
| Spironolactone | 1098 | 1.40 | 1.21-1.61 | <0.001 |  |  | 1.24 | 1.07-1.44 | 0.005 |  |
| Placebo | 1104 | 1.30 | 1.13-1.50 | <0.001 |  |  | 1.16 | 1.00-1.34 | 0.055 |  |
| **Cardiovascular death** |  |  |  |  |  |  |  |  |  |  |
| Region |  |  |  |  | 0.017 |  |  |  |  | 0.025 |
| Americas | 1005 | 1.03 | 0.88-1.21 | 0.710 |  |  | 0.97 | 0.81-1.15 | 0.730 |  |
| Russia/Georgia | 1197 | 1.47 | 1.15-1.87 | 0.002 |  |  | 1.42 | 1.10-1.84 | 0.008 |  |
| Gender |  |  |  |  | 0.360 |  |  |  |  | 0.530 |
| Male | 997 | 1.17 | 0.98-1.39 | 0.076 |  |  | 1.13 | 0.94-1.37 | 0.210 |  |
| Female | 1205 | 1.33 | 1.08-1.63 | 0.008 |  |  | 1.14 | 0.91-1.42 | 0.260 |  |
| Treatment group |  |  |  |  | 0.630 |  |  |  |  | 0.600 |
| Spironolactone | 1098 | 1.29 | 1.09-1.53 | 0.003 |  |  | 1.21 | 1.01-1.46 | 0.042 |  |
| Placebo | 1104 | 1.21 | 0.99-1.48 | 0.061 |  |  | 1.08 | 0.87-1.35 | 0.480 |  |
| **Any hospitalization** |  |  |  |  |  |  |  |  |  |  |
| Region |  |  |  |  | 0.001 |  |  |  |  | 0.006 |
| Americas | 1005 | 1.11 | 1.04-1.18 | 0.003 |  |  | 1.09 | 1.02-1.17 | 0.014 |  |
| Russia/Georgia | 1197 | 1.38 | 1.23-1.55 | <0.001 |  |  | 1.35 | 1.19-1.53 | <0.001 |  |
| Gender |  |  |  |  | 0.710 |  |  |  |  | 0.730 |
| Male | 997 | 1.30 | 1.20-1.41 | <0.001 |  |  | 1.25 | 1.15-1.36 | <0.001 |  |
| Female | 1205 | 1.32 | 1.22-1.44 | <0.001 |  |  | 1.19 | 1.08-1.31 | <0.001 |  |
| Treatment group |  |  |  |  | 0.960 |  |  |  |  | 0.820 |
| Spironolactone | 1098 | 1.32 | 1.21-1.43 | <0.001 |  |  | 1.22 | 1.12-1.32 | <0.001 |  |
| Placebo | 1104 | 1.32 | 1.21-1.43 | <0.001 |  |  | 1.22 | 1.11-1.34 | <0.001 |  |
| **HF hospitalization** |  |  |  |  |  |  |  |  |  |  |
| Region |  |  |  |  | 0.065 |  |  |  |  | 0.120 |
| Americas | 1005 | 1.04 | 0.93-1.16 | 0.540 |  |  | 1.03 | 0.92-1.16 | 0.600 |  |
| Russia/Georgia | 1197 | 1.54 | 1.02-2.33 | 0.039 |  |  | 1.52 | 1.00-2.31 | 0.052 |  |
| Gender |  |  |  |  | 0.780 |  |  |  |  | 0.430 |
| Male | 997 | 1.31 | 1.12-1.53 | 0.001 |  |  | 1.19 | 1.00-1.41 | 0.052 |  |
| Female | 1205 | 1.27 | 1.08-1.49 | 0.004 |  |  | 1.11 | 0.92-1.34 | 0.290 |  |
| Treatment group |  |  |  |  | 0.110 |  |  |  |  | 0.037 |
| Spironolactone | 1098 | 1.43 | 1.22-1.68 | <0.001 |  |  | 1.27 | 1.06-1.51 | 0.008 |  |
| Placebo | 1104 | 1.19 | 1.02-1.39 | 0.030 |  |  | 1.05 | 0.88-1.25 | 0.610 |  |

AF: atrial fibrillation, HR: hazard ratio, CI: confidence interval.

*Variables for adjustment in Region subgroups: gender, treatment arm, diabetes mellitus, smoke or ever smoke, body mass index, heart rate, diastolic blood pressure, estimated glomerular filtration rate; in Gender subgroups: treatment arm, diabetes mellitus, smoke or ever smoke, body mass index, heart rate, diastolic blood pressure, estimated glomerular filtration rate ; in Treatment subgroups: gender, diabetes mellitus, smoke or ever smoke, body mass index, heart rate, diastolic blood pressure, estimated glomerular filtration rate.


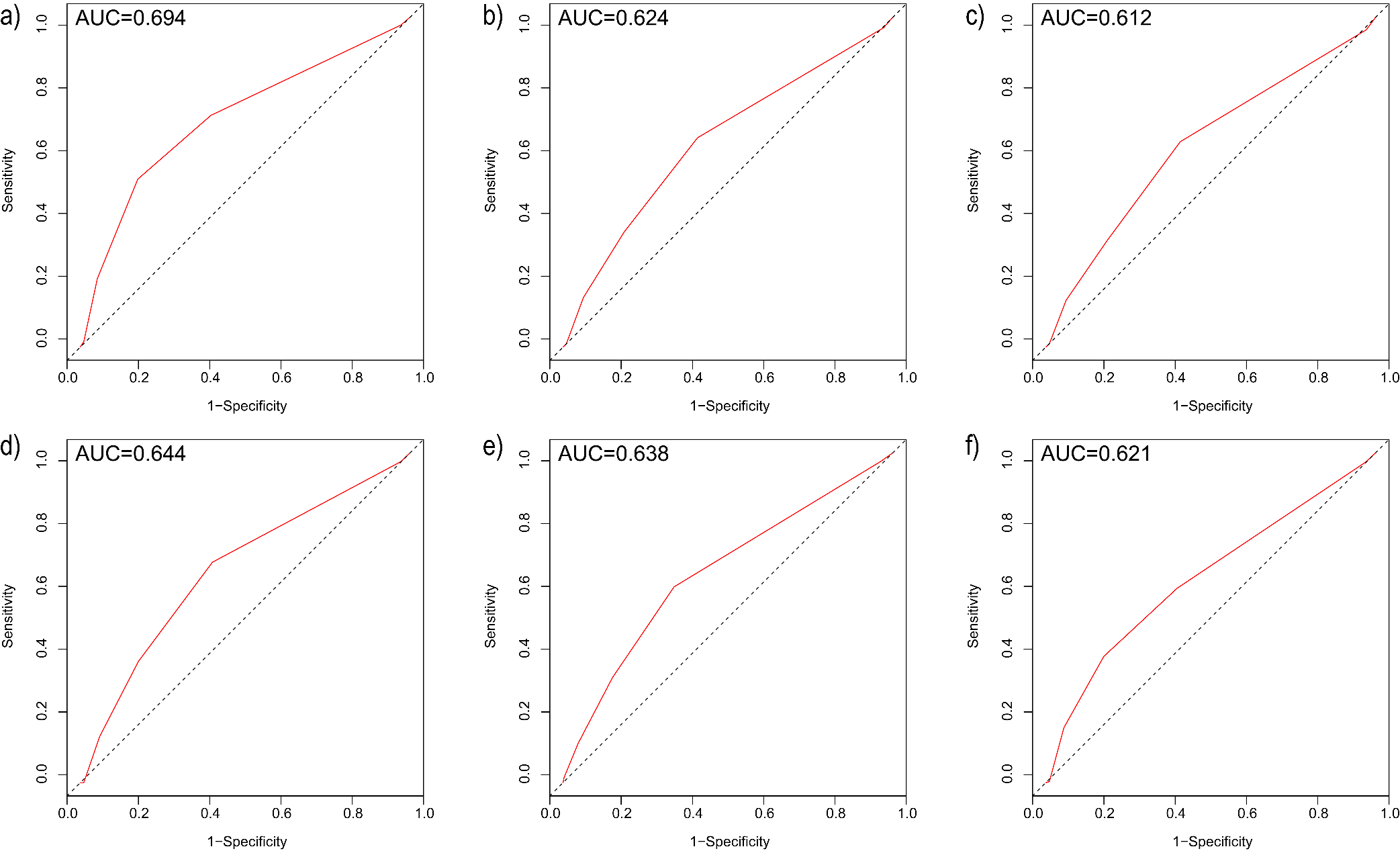


Fig. S1. Receiver operating characteristic (ROC) curves for the C_2_HEST score (with “HF” as a scoring item instead of “systolic HF”) in predicting a) atrial fibrillation, b) all-cause death, c) cardiovascular death, d) stroke, e) any hospitalization and f) heart failure hospitalization during follow-up.


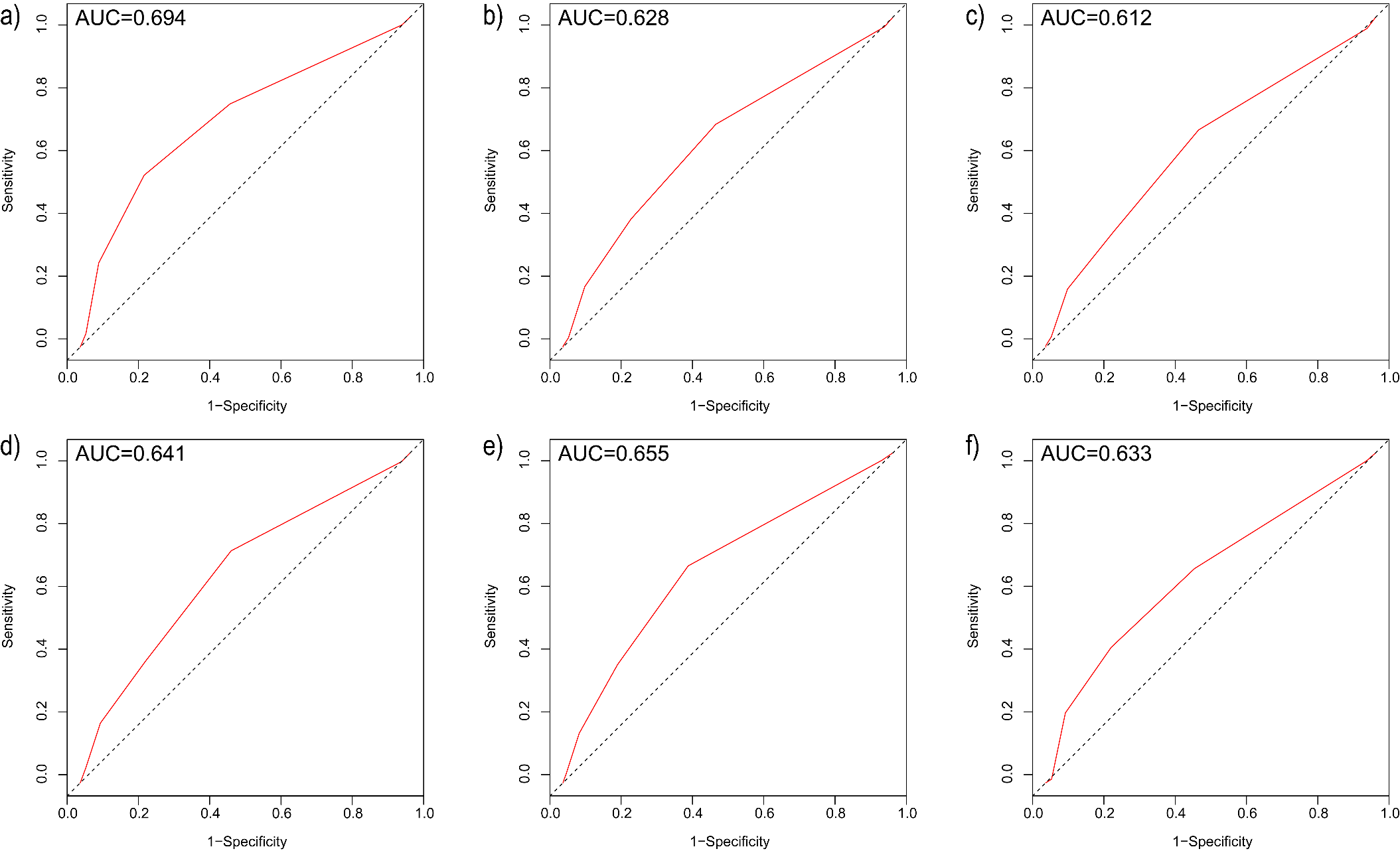


Fig. S2. Receiver operating characteristic (ROC) curves for the C_2_HEST score (with “hyperthyroidism” replaced by “thyroid disease”) in predicting a) atrial fibrillation, b) all-cause death, c) cardiovascular death, d) stroke, e) any hospitalization and f) heart failure hospitalization during follow-up.
